# Harmonic scalpel impact on blood loss and operating time in major head and neck surgery: a randomized clinical trial

**DOI:** 10.1186/s40463-016-0173-z

**Published:** 2016-11-08

**Authors:** Dieter K. Fritz, T. Wayne Matthews, Shamir P. Chandarana, Steven C. Nakoneshny, Joseph C. Dort

**Affiliations:** 1 Department of Surgery, Section of Otolaryngology – Head & Neck Surgery, Cumming School of Medicine, University of Calgary, HRIC 2A02, 3280 Hospital Dr. NW, Calgary, AB T2N 4Z6 Canada; 2 Ohlson Research Initiative, Arnie Charbonneau Cancer Institute, Cumming School of Medicine, University of Calgary, Calgary, Canada

**Keywords:** Oral cancer, Harmonic scalpel, Head and neck squamous cell carcinoma, Randomized clinical trial, Health technology assessment

## Abstract

**Background:**

Long operating time and high blood loss contribute to post-surgical morbidity. Therefore, strategies to reduce these factors should to be tested using robust methods.

The purpose of this study was to evaluate the impact of using the harmonic scalpel on operating time and blood loss in patients undergoing resection for advanced oral cancer (OSCC).

**Methods:**

Thirty-six adult head and neck cancer patients with advanced OSCC requiring primary tumor resection with uni- or bi- lateral selective neck dissection from July 2012 to September 2014 were randomized to either the control group (traditional surgery) or the experimental group (harmonic surgery). Patients older than 18 years who were able to provide informed consent were eligible. Primary outcomes of interest were: intraoperative blood loss (mL) and operative time (minutes) for the ablative part of the surgery.

**Results:**

Mean blood loss in the experimental group was 260 mL versus 403 mL in the control group (*p* = 0.08). Mean operative time was 140 min in the experimental group and 159 min in the control group (*p* = 0.2).

**Conclusions:**

In this randomized controlled trial, use of the harmonic scalpel did not effect intraoperative blood loss or OR time in patients undergoing surgery for advanced OSCC.

**Trial registration:**

ClinicalTrials.gov, NCT02017834.

## Background

Oral squamous cell cancer (OSCC) is among the top ten most prevalent malignancies affecting patients worldwide [1]. When advanced, treatment consists of a multidisciplinary approach involving the head and neck surgeon, radiation oncologist and medical oncologist. Surgical resection is the primary treatment of OSCC and can involve both complex resection and reconstruction. Surgical blood loss and prolonged operating time adversely impact treatment outcomes in a variety of surgical procedures including major head and neck surgery [2–5]. Neck dissection, either uni or bilateral is usually part of the treatment of advanced OSCC. Major head and neck resections are commonly performed using a variety of instruments including sharp dissection and electrocautery as well as suture ligatures and surgical clips for additional hemostasis. These common approaches have stood the test of time but have a number of potential disadvantages. These disadvantages include lost time due to frequent instrument passing for tissue cutting and hemostasis, increased local thermal tissue damage with electrocautery, supplementary clipping/suture ligatures for hemostasis of larger vessels and overall decreased operative efficiency and potential for increased blood loss.

The harmonic scalpel (HS)(Ethicon Endo-Surgery, Cincinnati, OH) was initially designed and utilized for laparoscopic surgery nearly two decades ago. Since that time, the HS has been adapted for use in a broad range of head and neck surgical procedures including tonsillectomy, thyroidectomy, glossectomy and submandibular gland excision [6–14]. The HS reduces intraoperative blood loss in thyroidectomy and reduced blood loss and operative time in parotidectomy [6, 9–13, 15]. As a result, many surgeons preferentially use the HS in order to improve surgical efficiency and reduce surgical bleeding.

Previous studies comparing the HS to standard techniques in selective neck dissection (SND) alone revealed a significant decrease in operative time, blood loss or both [16–20]. A recent randomized clinical trial from our group concluded that the HS is an effective tool for reducing blood loss in patients undergoing level I-IV neck dissection [16]. There are no randomized studies that measure the utility of the HS for oral resection combined with neck dissection. The addition of an oral resection with neck dissection has greater associated blood loss; therefore the HS may prove to be of particular benefit for these extended procedures.

The purpose of this study was to evaluate, using a prospective randomized design, the impact of the HS on blood loss and operating time for the surgical treatment of advanced OSCC. Our hypothesis was that the use of the HS will reduce both operative blood loss and time.

## Methods

This study was a prospective randomized clinical trial (www.clinicaltrials.gov registration #NCT02017834) undertaken in a consecutive cohort of patients presenting to the senior surgeons (T.W.M., S.P.C., J.C.D.). To be eligible, patients had to be 18 years old and have advanced oral cavity squamous cell carcinoma (clinical stage T2 or greater) requiring resection with unilateral or bilateral SND (I-IV). Patients were excluded if they received previous treatment for head and neck cancer, were unwilling or unable to consent to surgery or had a history of a bleeding disorder. Between July 2012 and September 2014, 36 consecutive patients presenting with a diagnosis of OSCC were eligible for this study. All patients provided informed consent to participate in this trial prior to their enrolment and allocation. This study was reviewed and approved by the Conjoint Health Research Ethics Board at the University of Calgary.

**Fig. 1:**
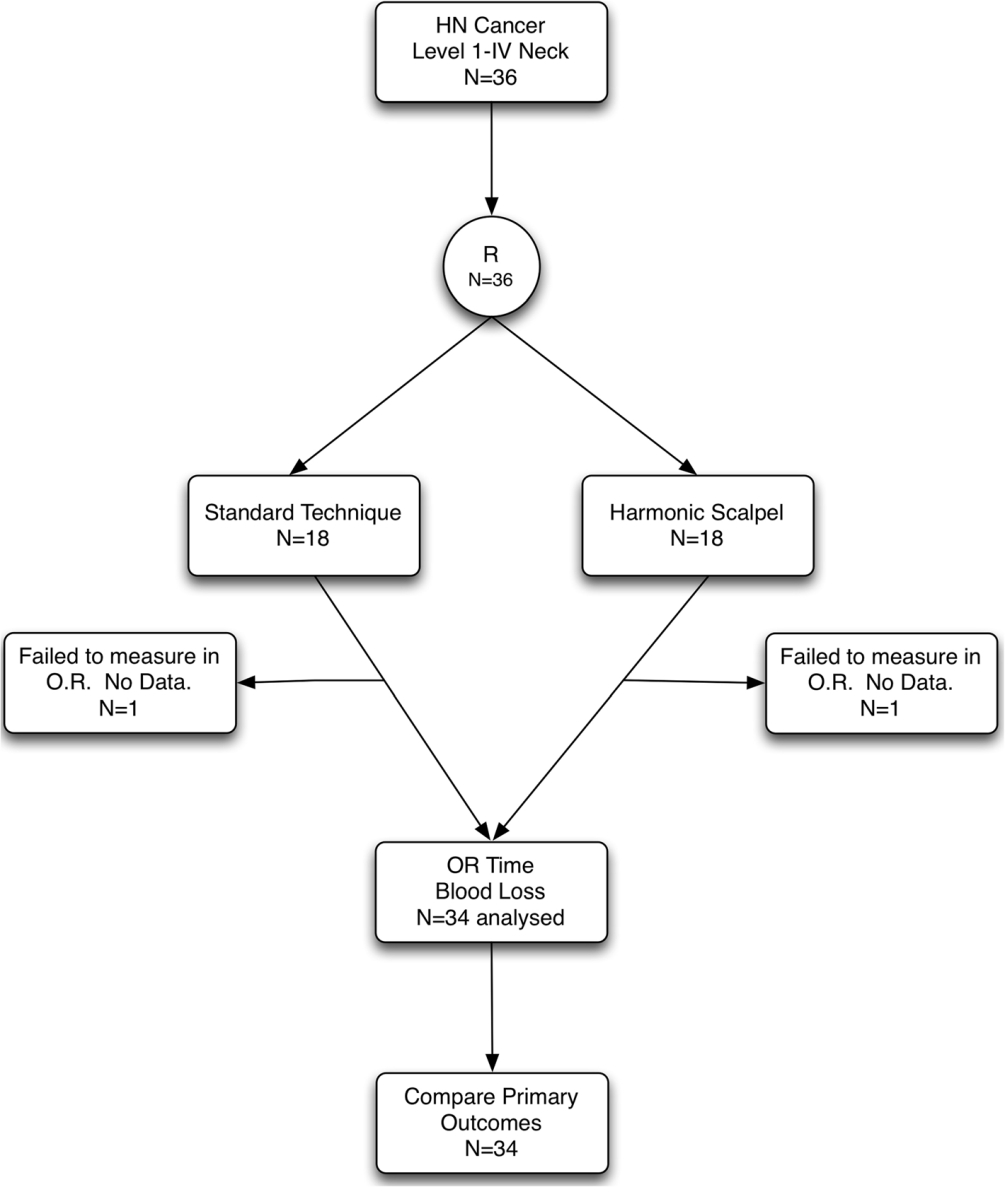
Study flow diagram. HN, head and neck; OR, operating room

Subjects were assigned to either the control or experimental groups via a predetermined 6 × 6 block randomization. The random allocation sequence was generated by two authors (J.C.D, S.C.N.) using randomization.com. Subjects were enrolled at the pre-operative consultation by one of the senior surgeons (T.W.M, S.P.C., J.C.D.). Patients were assigned sequentially to either arm of the study in accordance with the block randomization by the research coordinator (S.C.N), who informed the resecting surgeon one day prior to the OR. The control group (traditional surgery) comprised 18 combined oral resections and neck dissections in which our standard dissection technique (sharp dissection using scalpel or cutting cautery, surgical ties and/or clips for hemostasis augmented with bipolar and/or monopolar cautery) was used. The experimental group (harmonic surgery) consisted of 18 combined oral resections and neck dissections performed using the HS as an adjunct to our standard dissection technique. Of the 36 patients, the intraoperative outcomes were improperly measured for 1 patient in each of the experimental and control groups and they were therefore excluded from analysis as protocol violations. The remaining 34 combined procedures followed the full study protocol with no further exclusions, dropouts or protocol violations. The study flow diagram can be seen in Fig. 1.

### Study protocol

The following protocol was adapted from our previous work with the HS in neck dissection [16]. All patients were operated on by 1 of 3 experienced head and neck oncologic surgeons (T.W.M., S.P.C., J.C.D.) at the Foothills Medical Centre in Calgary, Alberta, Canada. Before being permitted to enroll patients to the study protocol, surgeons were required to individually perform 10 combined cases using the HS in order to become sufficiently proficient in the use of the device. Preoperative characteristics for all enrolled subjects including age, sex, body mass index (BMI), location of primary, cancer staging (TNM) and their Charlson Comorbidity Index score were collected at the time of enrollment.

At the time of surgery, the patient was positioned, prepped and draped using standard protocol. Once the tracheostomy was performed, the primary lesion and SND incisions were marked and then injected with 0.25 % Marcaine and 1:100,000 epinephrine. Subplatysmal skin flaps were raised prior to recording operative time and blood loss. Standard bipolar and monopolar cautery (Valleylab, Inc, Boulder, Colorado) were used in the traditional surgery patients. In the harmonic surgery patients, the Harmonic Focus hand piece (Ethicon Endo-Surgery, Cincinnati, Ohio) was used to perform the surgical resection. The operating surgeon also selectively used surgical clips, bipolar and monopolar cautery when the neurovascular anatomy precluded the use of the harmonic scalpel. All other surgical instruments were identical between the 2 groups. Blood loss was measured as the combined total of the volume of drainage in the suction canister and the weight of the sponges used (minus the dry weight of the sponges and any irrigation used). Operative times were recorded independently for each SND performed and primary tumor resection and then totaled. Resident operating time and experience as potential confounders were mitigated through the recording the percentage of the total time that the resident was operating in addition to the resident’s post-graduate training year.

In the post-operative period, the secondary outcome variables were collected at 48 h and at 1 week. These variables included: post-operative complications (Clavien complication scale), cumulative drain outputs (in milliliters), the presence/absence of abnormalities around the incision site (infection, hematoma, seroma) and hospital stay (in days). Neck drains were removed when the 24-h drainage was less than 25 mL – all drains were left for a minimum of 72 h. Completion of the protocol occurred at the 1-month period when the presence or absence of complications as well as the state of the incision site were recorded at a scheduled follow-up visit. The trial was concluded when the enrolment objective was acheived.

### Statistical analysis

Power and sample size were calculated based on the two primary outcomes of interest (blood loss, operating time) using a comparison of means of two independent samples (Stata, version 14. Stata Corp. College Station, Tx, USA). A sensitivity analysis using our own estimates of OR time and blood loss (based on clinical experience and review of the literature) was performed [12]. The sensitivity analysis and power calculation revealed that a sample size of 13 subjects per arm would allow us to detect a difference in operative time greater than 20 min and a difference in blood loss of greater than 200 ml (power 0.9, alpha 0.05). These differences were judged to be clinically meaningful. In order to allow for potential study dropouts and protocol violations we elected to recruit a total of 36 subjects (18 subjects per arm).

Intergroup differences for the primary outcomes of interest were compared using a nonparametric test for independent continuous outcomes (Wilcoxon Rank-sum test). Categorical variables were compared using a chi-squared test. A *p*-value < 0.05 was considered statistically significant.

## Results

All patients enrolled in the study underwent resection of an advanced OSCC (clinical T2 or greater) in addition to a unilateral or bilateral SND. Clinicopathologic characteristics of the cohort can be seen in Table 1. The groups were homogeneous for age, sex, BMI, TNM staging and comorbidity. Pathologic T and N stages are reported in Table 1.

**Table 1:** Patient demographics

	Harmonic scalpel (*n* = 17)	Traditional (*n* = 17)	*p*-value
Age at surgery			ns
yrs (mean, SD)	61 (9.2)	62 (12.9)	
Sex (*n*, %)			ns
Male	11 (65 %)	9 (53 %)	
Female	6 (35 %)	8 (47 %)	
BMI			ns
(mean, SD)	25.7 (0.8)	25.2 (1.4)	
pT stage (*n*, %)			ns
T1	2 (12 %)	1 (6 %)	
T2	7 (41 %)	4 (24 %)	
T3	3 (18 %)	1 (6 %)	
T4a	5 (29 %)	11 (65 %)	
pN stage (*n*, %)			ns
N0	7 (41 %)	8 (47 %)	
N1	6 (35 %)	4 (24 %)	
N2b	4 (24 %)	3 (18 %)	
N2c	0	2 (12 %)	
Overall stage (*n*, %)			ns
I	2 (12 %)	1 (6 %)	
II	2 (12 %)	1 (6 %)	
III	6 (35 %)	1 (6 %)	
IV	7 (41 %)	14 (82 %)	

### Primary outcomes

**Fig. 2:**
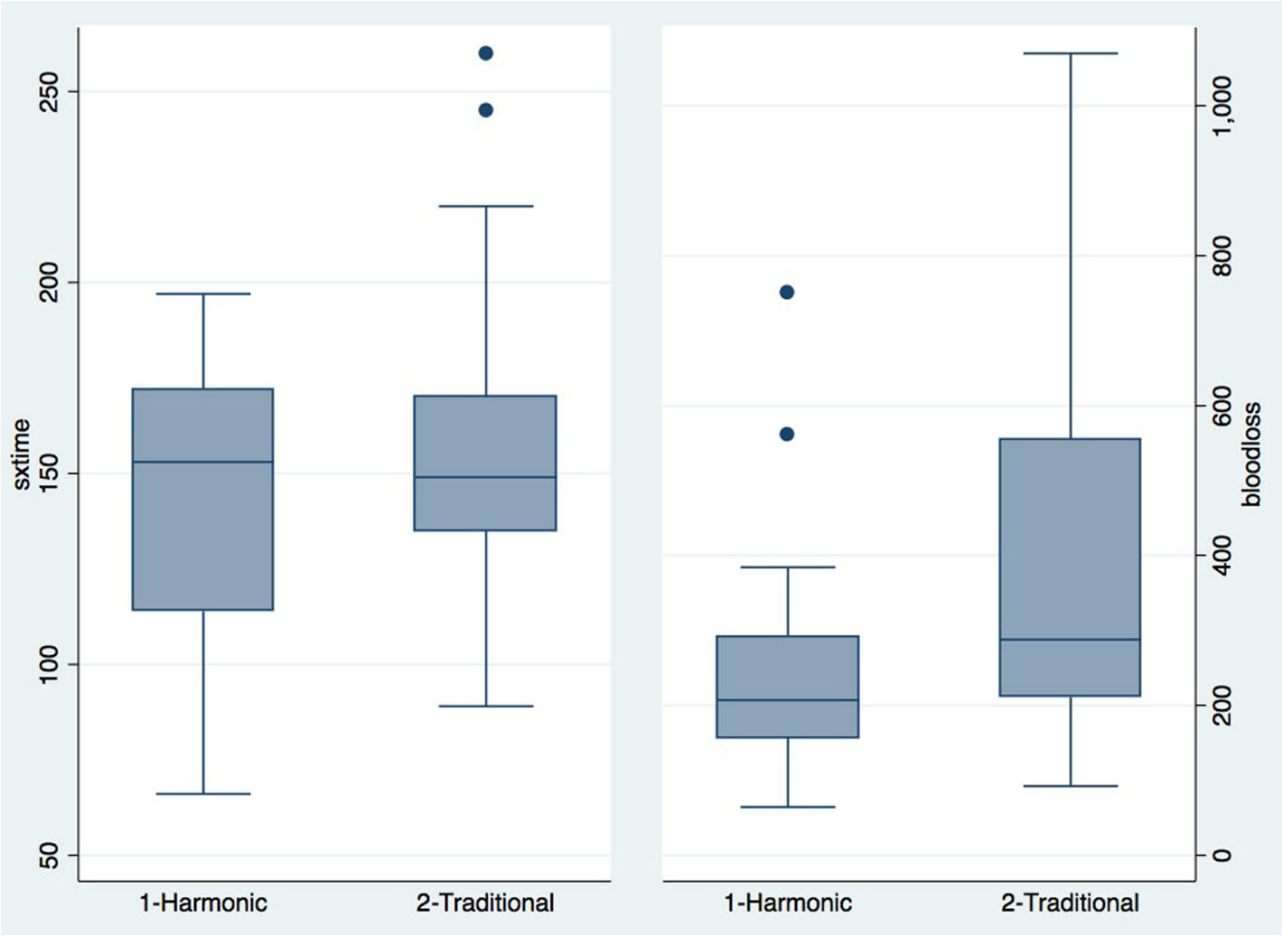
Primary outcomes

A comparison of the primary outcomes can be seen in Table 2 and Fig. 2. Intraoperative blood loss was not significantly lower in the experimental group compared to the control group (260 mL vs. 403 mL, *p* = 0.08). Two patients in the experimental group were extreme outliers in terms of blood loss. The mean total operative time was 140 min in the experimental group and 159 min in the control group (*p* = 0.21). Two subjects were also outliers with respect to OR time.

**Table 2:** Primary outcomes

	All patients			Excluding extreme outliers		
	Harmonic scalpel (*n* = 17)	Traditional (*n* = 17)	*p*-value	Harmonic scalpel (*n* = 15)	Traditional (*n* = 15)	*p*-value
Operative time, min (mean, 95 % CI)	140 [120–161]	159 [134–184]	ns	140 [119–161]	147 [127–166]	ns
Intraoperative blood loss, mL (mean, 95 % CI)	260 [171–349]	403 [261–545]	ns	207 [158–257]	403 [261–545]	0.01

Wilcoxon Rank-sum Test

### Secondary outcomes

There were 2 vascular injuries (internal jugular vein), 1 in each group. One patient in the harmonic group had a tumor that required resection of the hypoglossal nerve and this was recorded as a neurologic complication. There were no intraoperative adverse events. In the post-operative period, there were no surgical site complications (seroma, hematoma, wound infection). Other secondary outcomes are reported in Table 3. There were no differences between the control and experimental groups.

**Table 3:** Secondary outcomes

	Harmonic scalpel (*n* = 17)	Traditional (*n* = 17)	*p*-value
Intraoperative complications			
Vascular complications	1	1	ns
Neurologic complications	1	0	ns
Surgical drain output			
48-h, mL (mean, 95 % CI)	160 [107–213]	119 [84–154]	ns
1-week, mL (mean, 95 % CI)	214 [142–285]	185 [102–268]	ns
Hospital stay, days (mean, 95 % CI)	14 [11–18]	15 [12–18]	ns

## Discussion

In this randomized controlled trial we conclude that the harmonic scalpel had no significant impact on blood loss or operating time in patients undergoing oral cancer resection combined with neck dissection. Two patients in the harmonic scalpel group had extreme blood loss that was not explained by any evident surgeon, tumor or patient factors.

Tirelli et al. [21] performed a non-randomized trial evaluating the benefits and disadvantages of the HS in the treatment of 36 patients with oral and oropharyngeal carcinomas. These authors found a highly significant decrease in blood loss in the HS group, but did not differentiate between the oral and oropharyngeal sub-groups. There was also a significant reduction in OR time in the harmonic scalpel group. Oral and oropharyngeal cancer surgery are two distinct entities that can require a number of different surgical approaches and subsequently different amounts of surgical time and operative blood loss. The only disadvantage found by Tirelli et al was a higher incidence of lymphoedema in the HS group attributed to complete lymphatic interruption upstream to dissection. No other difference in complications was found between the two groups. The Tirelli study and ours are not directly comparable due to the differences in tumour sites and study design.

Our group [16], and others, have demonstrated the HS to be an effective tool in reducing operative blood loss in neck dissection. In this study the addition of the oral cavity resection seems to minimize the impact of the HS. Examination of the raw data revealed that the 2 outliers in the HS group strongly influenced the results and perhaps a larger study might show a beneficial effect of the HS on blood loss. However, our study was adequately powered to detect the magnitude of difference it set out to and the results are therefore valid. Similar to our previous study, we did not find a significant reduction in operative time when using the HS. This finding is in contrast to other studies that suggest that the HS is effective in reducing OR time. We cannot explain the discrepancy between our findings and others but we are confident that our prospective randomized study design is robust.

Despite the randomized study design we believe there are some limitations to this research. This was a single institutional study with 3 experienced head and neck surgeons performing advanced oral cancer resections and neck dissections. This potentially limits the generalizability of the findings. A multicenter study could reduce any surgical technique bias produced by evaluating surgeons from a single center. Our experience using the HS in oral resection suggests that it might be a useful tool in reducing blood loss in some patients but it is by no means a panacea that should be universally applied.

## Conclusions

In conclusion, our study is the first randomized trial to evaluate the harmonic scalpel in the treatment of advanced OSCC. Despite our clinical impression, we did not find significant differences in OR time or blood loss between the 2 treatment groups. A multicentre clinical trial would be a useful next step in determining the true value of the harmonic scalpel in major head and neck resection.
